# Mechanisms of Connexin Regulating Peptides

**DOI:** 10.3390/ijms221910186

**Published:** 2021-09-22

**Authors:** D. Ryan King, Meghan W. Sedovy, Xinyan Leng, Jianxiang Xue, Samy Lamouille, Michael Koval, Brant E. Isakson, Scott R. Johnstone

**Affiliations:** 1 Fralin Biomedical Research Institute at Virginia Tech Carilion School of Medicine, Virginia Tech, Roanoke, VA 24016, USA; ryanking@vt.edu (D.R.K.); msedovy@vt.edu (M.W.S.); xleng@vt.edu (X.L.); lamouils@vt.edu (S.L.); 2 Translational Biology, Medicine, and Health Graduate Program, Virginia Tech, Blacksburg, VA 24061, USA; 3 Robert M. Berne Cardiovascular Research Center, University of Virginia School of Medicine, Charlottesville, VA 22908, USA; uxm8hs@virginia.edu (J.X.); brant@virginia.edu (B.E.I.); 4 Center for Vascular and Heart Research, Virginia Tech, Roanoke, VA 24016, USA; 5 Department of Medicine, Division of Pulmonary, Allergy, Critical Care and Sleep Medicine, Emory University School of Medicine, Atlanta, GA 30322, USA; mhkoval@emory.edu; 6 Department of Molecular Physiology and Biophysics, University of Virginia School of Medicine, Charlottesville, VA 22908, USA; 7 Department of Biological Sciences, Virginia Tech, Blacksburg, VA 24060, USA

**Keywords:** connexin, gap junction, hemichannel, pannexin, peptide, cell signaling

## Abstract

Gap junctions (GJ) and connexins play integral roles in cellular physiology and have been found to be involved in multiple pathophysiological states from cancer to cardiovascular disease. Studies over the last 60 years have demonstrated the utility of altering GJ signaling pathways in experimental models, which has led to them being attractive targets for therapeutic intervention. A number of different mechanisms have been proposed to regulate GJ signaling, including channel blocking, enhancing channel open state, and disrupting protein-protein interactions. The primary mechanism for this has been through the design of numerous peptides as therapeutics, that are either currently in early development or are in various stages of clinical trials. Despite over 25 years of research into connexin targeting peptides, the overall mechanisms of action are still poorly understood. In this overview, we discuss published connexin targeting peptides, their reported mechanisms of action, and the potential for these molecules in the treatment of disease.

## 1. Introduction

### Gap Junctions, Hemichannels, and Connexin Proteins as Therapeutic Targets

Gap junction channels (GJ) were first identified in the 1960s [1,2,3] and have since been reported to control numerous physiologic and pathophysiologic functions. GJs are composed of connexin (Cx) proteins, of which 21 isoforms have been identified [4]. All known connexin isoforms are structurally similar, being composed of 4 transmembrane domains, 2 extracellular loops (EL1, EL2) bound in conformation by extracellular disulfide bonds, a single intracellular loop (IL), an amino-terminus (NT), and a carboxyl-terminus (CT) [4,5]. Variations among connexin isoforms are primarily found within the length and amino acid sequence of the IL and CT domains. Connexin proteins hexamerize to form single membrane channels called hemichannels (also known as connexons) in either the endoplasmic reticulum or the Golgi apparatus, depending on isoform, prior to trafficking to the plasma membrane [6]. Once at the membrane, hemichannels may dock with compatible hemichannels on the membrane of neighboring cells, forming GJs that permit direct intercellular cell-to-cell signaling.

GJs are non-selectively permeable to molecules up to ~1 kDa and are not considered to be highly charge selective, although charge may play a role in altering rates of transfer of molecules across the pore due to steric hindrance [5,7,8,9,10]. Hemichannels have been shown to function independently of GJ formation, facilitating the release of molecules such as ATP, ADP, and glutamate, and regulating Ca^2+^ flux in adipocytes, astrocytes, and glioblastoma cells [11,12,13,14,15,16]. While hemichannel signaling has been canonically studied at the plasma membrane, recent evidence suggests that hemichannel signaling is also present at the mitochondrial membranes [17,18,19]. Connexin proteins, independent of channel formation (GJ or hemichannel), have also been shown to act directly as signaling molecules, forming protein-protein interactions that regulate cellular fate [20,21,22,23,24,25]. Further, internal translation of the Cx43 protein leads to the formation of short connexin isoforms (20–30 kDa, e.g., GJA1-20k) that function within the cell to facilitate protein trafficking to the plasma membrane, mitochondrial membranes, and nuclear compartment [26,27,28,29,30,31].

**Figure 1:**
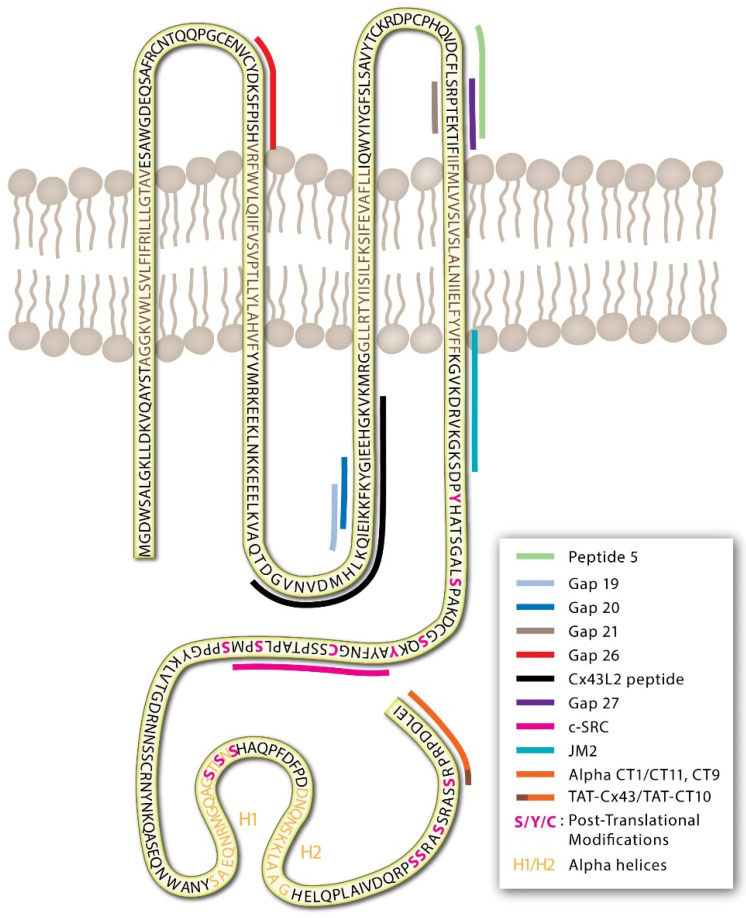
Schematic of the Cx43 protein in the plasma membrane with colored lines indicating the positions of described peptides targeting EL, IL, and CT regions.

GJs and connexins are dynamically regulated through protein post-translational modifications of the IL, NT, and CT [32,33,34,35,36,37,38,39]. The intracellular CT domain of most connexin proteins consists of a largely unstructured stretch of amino acids that tend to be rich in modifiable residues that can undergo nitrosylation (cysteine), phosphorylation (serine/threonine/tyrosine), and SUMOylation (lysine) as well as other modifications via acetylation, hydroxylation, and carboxylation (Figure 1) [33,40,41,42,43,44,45]. Post-translational modifications may lead to conformational changes in extracellular domains to inhibit channel docking and limit GJ formation, in pore-forming sites to alter channel permeability, and in signaling and intracellular domains that affect protein trafficking, membrane stability, and protein-protein interactions.

Connexin isoform expression and function are variable throughout the body. Dysfunctional GJs are often associated with diseases such as cancer, cardiovascular disease, wound and tissue remodeling, and sensoneurial defects [5,34,46,47,48,49,50]. As such, GJs and connexin proteins are attractive therapeutic targets. While there are presently no therapeutics directly targeting connexin proteins or GJs that are approved for clinical use, several have been investigated in clinical trials [51,52,53]. The primary focus for intervention has been peptides that have indirect and direct effects on the connexin channels and protein functions. There have been many excellent reviews on connexin therapeutics and peptides developed over the last 25 years [54,55,56,57,58,59]. In this review, we focus specifically on GJ and connexin peptides that have been developed and aim to discuss the potential mechanisms through which they function. This is an area of much debate and there has been a recent push towards understanding the peptide-protein structural relationship.

## 2. Indirect Acting Peptides That Alter GJs and Connexins

Cx43 has long been identified as a major cardiac connexin, regulating conduction between cardiomyocytes and playing critical roles in normal heart function through GJ and hemichannel signaling [60]. Reduced Cx43 expression, altered membrane localization, and changes in connexin phosphorylation states have all been observed during ischemic heart disease and are associated with the development of cardiac arrhythmias [61,62,63]. In 1980, a naturally occurring anti-arrhythmic peptide, labeled AAP10, was identified in bovine serum [58,64]. Given the links between Cx43 dysregulation and arrhythmia development, studies of the AAP10 peptide and its derivatives (Table 1) have centered around their effects in promoting GJ function and subsequent maintenance of cardiac rhythmic conduction [51,65]. 

As the identification of AAP10, more stable forms of anti-arrhythmic peptides have been manufactured [58]. From the original AAP10 molecule, modifications by sequence inversion and replacement by D-amino acids led to the production of ZP123 (rotagaptide), a more stable peptide analog [58]. Multiple pre-clinical trials in rat, rabbit, pig, and canine models found AAP10 and rotagaptide to preserve cardiac conduction, increase GJ signaling, and limit ischemia-induced cardiac damage and arrhythmia development [51,65,66,67,68]. Further modifications of the AAP10 led to the production of danegaptide (also referred to as ZP1609 and GAP-134), which showed a similar degree of reduction in post-ischemic infarct size in pig and canine models [69,70]. Despite the promising pre-clinical data for AAP10, rotagaptide, and danegaptide, none of these peptides were found efficacious in Phase II clinical trials [52,58]. The exact reason for a lack of efficacy is not clear but has been speculated elsewhere [58,71]. 

Despite their failure to decrease phenotypic disease burden, AAP10, rotagaptide, and danegaptide do alter the pattern of Cx43 expression, phosphorylation, GJ signaling, and hemichannel signaling [20,72,73,74]. Studies have demonstrated that these peptides increase both Cx43 and Cx40 GJ signaling [72,75]. Unlike the peptides to be discussed later in this review, the AAP10 derivative peptides do not contain sequence homology with connexin proteins. AAP10, rotagaptide, and danegaptide are not presumed to be membrane permeable. Rather, the peptides are suspected to bind with a membrane-bound G-protein coupled receptor (GPCR), although the precise receptor remains unknown [76,77]. It is hypothesized that peptide binding with a GPCR leads to the activation of protein kinase C (PKC) pathways, which in turn modify Cx43 and Cx40 functions [72,76]. Prolonged treatment with AAP10 and rotagaptide has been shown to increase Cx43 protein synthesis, expression, and membrane retention in cultured HeLa cells, primary rat ventricular cardiomyocytes, and dermal fibroblasts [20,72,78,79].

Phosphorylation is a key event in the lifecycle of most connexin proteins. For instance, in Cx43, de-phosphorylation of Cx43-S282 or phosphorylation at Cx43-S373 is linked to a reduction in GJ signaling following ischemia [61,80]. Conversely, phosphorylation at sites such as PKC-associated Cx43-S262 and Cx43-S368 are reportedly protective against ischemia-reperfusion injury [80,81]. In ischemic hearts, temporal reductions in site-specific Cx43 phosphorylation are associated with asystole, which is alleviated through treatment with AAP10 in rabbits and by rotagaptide in rats [65,68]. Both AAP10 and rotagaptide promote PKC activation, inducing Cx43-S368 phosphorylation and subsequent opening of Cx43 GJs [65,72,78,82,83,84]. Mass spectrometry analysis of cardiomyocytes also demonstrates that phosphorylation at S297 and S368 is lost subsequent to ischemia in untreated hearts, yet preserved by treatment with rotagaptide [58,68,85].

Studies in cultured endothelial cells suggest that danegaptide preserves Cx43 GJ signaling and reduces apoptosis under high glucose conditions [86]. In astrocytes, danegaptide treatments increase GJ and hemichannel signaling and produce protective effects such as reduced infarct volume following a stroke in mice [87]. In aged mice, danegaptide induces similar effects to AAP10 and rotagaptide, increasing Cx43-S368 phosphorylation, preserving Cx43 expression with the overall effect of reducing cardiac fibrosis [74]. In addition to membrane-associated GJ functions, these peptides were proposed to stabilize Cx43, which could limit the generation of damaging reactive oxygen species associated with mitochondrial Cx43. However, in murine cardiac cell preparations, danegaptide was found to exert its effects independent of mitochondrial Cx43, suggesting it may have other molecular targets and pathways outside of Cx43 [19,69,88]. 

The AAP10 peptide has also been proposed to enhance cancer therapeutic treatments [89], stem cell differentiation [90], and may increase homocysteine-induced cardiomyocyte cell apoptosis [91]. However, the exact pathways through which this occurs remain to be fully investigated, and more insight is required into how the anti-arrhythmic peptides function by binding GPCRs resulting in therapeutic protection.

**Table 1:** Connexin regulating peptides.

Year	Peptide	Sequence/Formula	Known Target Cx		Linker	Properties	Increase Cx43-PKC	Refs.
			Cx	Region				
**Non-Mimetic Peptides—Indirect Effects**								
1980	AAP10	H-GAG-4hyp-PY-CONH	Cx43 * Cx40 *	Indirect: Unknown GPCR pathway	-	Increases Cx synthesis, expression, phosphorylation, membrane targeting, and GJ opening	Y	[58,72,76,77,84]
2003	Rotagaptide (ZP123)	H2N-GDAGD-4hyp-DPDY-Ac	Cx43 *		NT Acetyl		Y	[51]
2013	Danegaptide (ZP1609, GAP134)	C_14_H_17_N_3_O_4_	Cx43 *		-		Y	[69,74]
**Extracellular Loop 1 (EL1)**								
1997	Gap26	VCYDKSFPISHVR	Cx43, Cx32, Cx26	64–76	-	HC block	Y	[92,93]
2001	^43^Gap26	VCYDKSFPISHVR	Cx43	64–76	-	HC block	Y	[94,95]
2009	^43^Gap26M	VCYDKSFPISHVR	Cx43	64–76	NT Acetyl	HC block	Y	[95,96]
2001	^37,40^Gap26	VCYDQAFPISHIR	Cx37, Cx40	64–76	-	HC block	ND	[94]
1999	Unlabelled	ICNTLQPGCNSV	Cx32	52–63	-	GJ block	ND	[97]
2007	Peptide 1848	CNTQQPCCENVCY	Cx43	54–66	-	GJ Block	ND	[98]
**Extracellular Loop 2 (EL2)**								
1999	Unlabelled	SLSAVYTCKRDPCPHE	Cx43	180–195	-	GJ block	ND	[99]
1999	Unlabelled	FLDTLHVCRRSPCPHP	Cx40	177–192	-	GJ block	ND	[99]
2001	^37,43^Gap27	SRPTEKTIFII	Cx43, Cx37, Cx32, Cx26	201–211	-	HC block	Y	[92,93,94,95,100]
2001	^40^Gap27	SRPTEKNVFIV	Cx40	201–211	-	GJ block	ND	[93,94,100]
2011	^32^Gap27	SRPTEKTVFT	Cx32	182–191	-	HC block	ND	[101]
2021	^62^Gap27	SRPTEKTIFML	CX62	201–211	-	HC & GJ block	ND	[102]
2008	Peptide 5	VDCFLSRPTEKT	EL2	EL2	s-lipidation	HC & GJ block	ND	[103,104,105,106]
2013	SRPTEKT/GAP21	SRPTEKT	Cx32 Cx43	182–188 204–210	-	HC block	ND	[92,101]
2018	SRPTEKT-Hdc (Gap21)	SRPTEKT-Hdc	Cx43	EL2	Hexadecyl (HC) lipid moeity	HC & GJ block	Y	[107,108]
2013	C12-Cx43 MP and C12-C12-Cx43 MP	C12-VDCFLSRPTEKT C12-C12-VDCFLSRPTEKT	Cx43	199–210	1/2 C12-Laa moieties	HC block	ND	[109]
**Intracellular Loop (IL)**								
2006	^32^GAP24 TAT-GAP24	GHGDPLHLEEVKC YGRKKRRQRRRGHGDPLHLEEVKC	Cx32 Cx43 Panx1	110–122	+/− TAT	HC block Inhibit GJ formation	ND	[10,110,111]
2004	L2 (Cx43L2)	DGVNVEMHLKQIEIKKFKYGIEEHGK	Cx43	119–142	-	HC block, not GJ GJ block	ND	[42,112,113]
2010	TAT-L2	TAT-DGANVDMHLKQIEIKKFKYGIEEHGK	Cx43	119–142	TAT	HC block	ND	[114]
2013	Gap19	KQIEIKKFK	Cx43	128–136	-	HC block	ND	[111,115,116]
2014	TAT-Gap19	KQIEIKKFK	Cx43	128–136	TAT	HC block	ND	[117]
2020	Xentry-Gap19	KQIEIKKFK	Cx43	128–136	LCLRPV	HC block		[118]
2010	TAT-Cx50L2	GGERAPLAADQGSVKKSSSSSKGTKK	Cx50	122–147	TAT	ND Cx50, No effect on Cx43 HC	ND	[114]
	Gap 20	EIKKFKYGC	Cx43	131–138	-	No effect	ND	[119]
	Gap 22	AELSCNKEVNG	Cx40	130–140	-	No effect	ND	[92]
**N-terminus/C-terminus**								
2005	Alpha CT1	RPRPDDLEI	Cx43	374–382	Antenna-pedia	Promotes GJ formation, enhance GJ, HC block	Y	[120,121,122]
2009	Alpha CT11 aka (Alpha CT2)	RPRPDDLEI	Cx43	374–382	None	Promotes GJ formation, enhance GJ, HC block	Y	[122,123]
2009	Alpha CT3	RQPKIWFPNRRKPWKKRPSSRASSRASSRPRPDDLEI	Cx43	359–382	Antenna-pedia	ND	ND	[122]
2010	TAT-Cx43CT	SRPRPDDLEI	Cx43	373–382	TAT	Maintains open channel and permits dye transfer. Inhibits HC block	ND	[114]
2011	CT9	RPRPDDLEI	Cx43	374–382	+/− TAT	HC block	Y	[101,124,125]
2013	TAT-CT10	SRPRPDDLEI	Cx43	373–382	TAT	Inhibits the effect of CxL2 peptides—stops L2 hemichannel blockade	ND	[115]
2010	TAT-Cx50CT	SRARSDDLTV	Cx50	431–440	TAT	ND–Cx50, No effect on Cx43 HC	ND	[114]
2010	ZP2519	AcRRK-(4 hydroxy benzoyl)	Cx43 C-term	-	-	GJ opening	ND	[126]
2015	Juxtamembrane 2 (JM2)	VFFK-GVKDRVKGRSD	Cx43	231–245	Antenna-pedia	HC block	ND	[127]
2020	TAT-Cx43 266–283	AYFNGCSSPTAPLSPMSP	Cx43	266–283	TAT	ND	ND	[128,129]

* indicates alterations through a GPCR pathway. Abbreviations used in the table: Cx—connexin; GJ—gap junction; HC—hemichannel; GPCR—G protein-coupled receptor; ND—not demonstrated; CT—carboxyl-terminus; EL—extracellular loop; IL—intracellular loop; NT—amino-terminus.

## 3. Connexin-Mimetic Peptides

Connexin-mimetic peptides differ from the AAP10 peptide derivatives in that they represent consensus amino acid sequence alignments for EL (1/2), IL, or CT regions of connexins (Figure 1). Connexin-mimetics initially were synthesized for use as epitopes for connexin antibody production [130,131]. The blocking antibodies raised using these peptides against the EL and IL domains of several connexins were tested as GJ blockers and demonstrated some effect in reducing dye transfer in chick embryos [116]. However, EL loop antibodies Gap7M (EL1/2), Gap11 (EL1/2 Cx32), Gap15 (IL of Cx43), and Gap17 (CT Cx40) were found to be ineffective in functional testing and were not able to reduce connexin-associated contractile responses in rabbit arterial tone [92]. Despite this, Dahl et al. described that peptides, used to generate antibodies, were effective in reducing Cx32 GJ formation and signaling in oocytes [132]. In 1995, Becker et al. found that connexin-mimetic peptides, mimicking the sequences of the IL domain, could effectively block GJ channels when directly injected into embryo cells [116]. In the subsequent decades, a number of connexin-mimetic peptides have been generated and tested with a multitude of proposed functions including blockade of hemichannel and GJ signaling, alterations in GJ formation, and disruption of protein localization and protein-protein interactions (Table 1). While a few pathways of action have been identified, many of these peptides still function through unknown mechanisms.

### 3.1. Connexin-Mimetic Peptides That Target the Extracellular Loops

Connexin targeting peptides were initially designed with sequence homology to the EL regions of the protein, with the intent of direct peptide-protein binding and subsequent GJ channel blockade [54,92]. Exactly why the connexin-mimetic peptides would bind in this way has not been well described, but it was assumed that they could act to inhibit normal protein interactions or docking functions of hemichannels to GJs, thus blocking hemichannel opening, GJ formation, and/or GJ signaling [54,123,133].

The early peptides targeting the EL—Gap21, Gap26, Gap27, and “Peptide 5” (a peptide shifted by 5 amino acids towards the amino-terminus of Gap27)—were designed against EL1 and EL2 of Cx32 and Cx43 (Table 1) [92,134]. Peptides targeting the EL of Cx32 (Gap21) were shown to delay GJ formation and electrical synchronization in chick cardiomyocytes [134]. Gap26 and Gap27 peptides effectively reduce Cx43 GJ and hemichannel functions [135,136]. Peptide 5, described by O’Carroll et al. [103], is reported to block hemichannel functions with effects in neuroprotection following spinal cord injuries, vascular leak, and ischemia [104,105,106,137]. In rabbit arterial sections, Gap21, Gap26, and Gap27 reduce phenylephrine-induced vasoconstriction [92].

Given the proposed consensus peptide-protein binding mechanism, it was presumed that the connexin-mimetics would be highly specific. For example, the Gap26 peptide sequence, VCYDKSFPISHVR, corresponds to amino acids 64–76 of Cx43-EL1. However, this peptide also altered the channel functions of Cx32, Cx37, and Cx40 in a wide range of tissues [54]. This appears to result from the highly conserved nature of connexin sequences in the EL regions [94,96,138]. For example, Gap26 has 100% homology with human Cx43-EL (accession CAG46461), but also greater than 75% similarity with Cx40-EL (accession NP_005257), Cx32-EL (accession NP_001091111), and Cx37-EL (accession number NP_002051.2). Studies by Warner et al. suggested that key peptide amino acid motifs SHRV and SRPTEK, found in many connexin EL1 and EL2 loops, may play a dominant role in channel blockade [92,134]. It is possible then, that these sites are critical for peptide-protein binding, which potentially limits connexin specificity. 

Peptides that are reportedly more specific, targeting Cx32, Cx37, Cx40, and Cx43, were later developed by adjusting the amino acid sequences within Gap26 and Gap27 to produce ^37,40^Gap26, ^43^Gap26(M), ^32^Gap27, ^40^Gap27, and ^37,43^Gap27 (Table 1) [94]. Most recently, ^62^Gap27 has also been designed and tested for the inhibition of Cx62 in platelets, with no reported cross-talk with Cx40 and Cx37 hemichannels [102]. ^43^Gap26 and ^37,43^Gap27 have wide-ranging effects reported in many tissues including the inhibition of acetylcholine responses in smooth muscle cells, reduced dye transfer in cultured cells [139], accelerated migration and wound closure in fibroblasts, keratinocytes, and epithelial cells [46,140,141], and limited ischemic damage following injury in hearts [142]. The design of ^40^Gap27 only differs from the ^37,43^Gap27 by three amino acids and still contains the SRPTEK motif [93,94,100,143]. Several studies suggest that ^40^Gap27 is specific to Cx40 GJs but not Cx43 or Cx37 [93,100,139]. ^40^Gap27 does not inhibit dye transfer in COS-7 fibroblasts that express Cx43 and not Cx40, demonstrating that the ^40^Gap27 peptide does not regulate Cx43 [93,100]. The addition of lipid alkyl groups (C12-Laa) increased peptide stability of a short region of the GAP27 peptide by around 2 fold [109]. This was further adapted by incorporating a hexadecyl lipid moiety (C16 lipid, hexadecyl-NH2; Hdc) at the CT of “Peptide 5” and GAP27 peptides to produce SRPTEKT-Hdc [107,108]. SRPTEKT-Hdc is reported to block hemichannels and GJs, as measured by inhibition of hemichannel dye uptake (opening) and reduced Ca^2+^ wave propagation [107,108].

Gap26 and Gap27 peptides, and their iterations, have been shown to increase intracellular Ca^2+^ concentrations, similar to observations following treatments with the non-selective GJ blocker glycyrrhetinic acid in cultured endothelial cells and smooth muscle cells [100,101]. The peptides desynchronized Ca^2+^ transients in cultured cells and rat mesenteric arteries associated with reductions in vasomotion [101,144]. In cultured endothelial cells and smooth muscle cells, ^37,40^Gap26 and ^40^Gap27 inhibited GJ-based dye transfer, but did not alter synthesis or de novo formation of GJ plaques at concentrations up to 600 μM [100]. However, later studies suggested that this may be cell type-specific, as Gap27 decreases transcription and protein expression of Cx43 in juvenile dermal fibroblasts [46]. 

Both Gap 26 and Gap27 peptides induce PKC phosphorylation of Cx43-S368, associated with reduced GJ signaling [95,108,145,146]. The EL2 peptide SRPTEKT-hdc (containing the same Cx sequence as Gap21 [92]) blocked GJ function, which is also related to increased PKC-associated Cx43-S368 phosphorylation [107,108]. Treatments with ^62^Gap27 also activate protein kinase A (PKA) pathways in platelets, although it is not currently clear if this has a direct effect on the Cx62 protein phosphorylation [102].

It is still not clear how the EL-targeting peptides function. To our knowledge, no conclusive studies have shown direct binding of the EL-targeting peptides with their corresponding connexin sequences. A recent blind docking molecular mechanics modeling approach studying Gap26, Gap27, and “Peptide 5” suggests that the peptide binding site may be unrelated to the connexin-mimetic sequence [147]. Simon et al. suggested that, due to a number of factors including a lack of access to the sites, binding most likely occurs within the inner EL1 surface and EL1-2 interface [147]. Wang et al. also suggested that peptide size may play a role in the inhibition of connexin channels, potentially leading to steric hindrance of the channel [10], although this has been debated for other peptides [115].

The idea of channel block in this manner has been discussed in detail [138], but there is little direct evidence for this mechanism. Gap26 and Gap27 peptides are around 1300–1500 Da in size, and there is evidence to suggest that peptides up to 1800 Da can enter via connexin hemichannels [148]. This leads to the possibility that EL peptides can target protein sites distinct from the EL regions as other connexin-mimetic peptides have been found to bind multiple regions of the same connexin protein [123]. 

### 3.2. Connexin Mimetic Peptides That Target the Intracellular Loop

A model for the control of GJ (Cx43 and Cx40) channel gating was suggested in studies by Delmar and colleagues, where they described a “ball-and-chain” gating mechanism [149,150,151,152]. Their model identified a pH-dependent interaction of the flexible connexin-CT with a portion of the connexin-IL [149,151,153]. Based on these studies it was proposed that the connexin-IL acts as a receptor site for regulation of the GJ pore, in that, binding at this site by connexin-CT leads to closure of the channel [154]. Using protein interaction studies (ELISA, NMR) based techniques, Delmar et al. identified a connexin-mimetic peptide of the Cx43-IL that binds the Cx43CT, later described as the L2/Cx43L2 peptide (Table 1) [42]. In vitro studies of purified proteins demonstrated that the Cx43L2 peptide and Cx43-CT directly interact in a pH-dependent manner [42]. 

There is very little sequence homology in the connexin-IL, unlike the -EL regions, therefore peptides are presumably isoform-specific. Early peptide studies targeting the Cx43-IL, using peptides within the L2 region, GAP 13 and GAP 15 peptides, required direct injection of the peptides into cells to produce a functional block, due to the inability of the peptide to cross the plasma membrane [116]. Since then, many studies have linked cell-penetrating moieties that permit the membrane translocation of peptides. The most commonly used forms of these in connexin-mimetic peptide studies has been the HIV-TAT sequence (YGRKKRRQRRR) [153,155], antennapedia sequence (RQPKIWFPNRRKPWKK) [156], and the cell-penetrating peptide sequence (LCLRPV) derived from the X-protein of hepatitis B virus [118], added to the NT or CT of each peptide (Table 1). Several studies have demonstrated the effectiveness of these approaches in permitting the internalization of the connexin mimetic peptides [114,117,121].

To test the functions of the IL-CT interaction in the role of GJ coupling or hemichannel signaling outside of structural studies, several other L2 peptides were developed by Leybaert and colleagues with an attached TAT sequence [114]. In these studies, the authors developed TAT-bound peptides for the Cx50L2 (TAT-Cx50L2) as well as CT peptides for Cx43 and Cx50 (TAT-Cx43CT, TAT-Cx50CT, Table 1). TAT-Cx50L2 peptides produced no effect [114], and the function of the TAT-Cx43CT/-Cx50CT peptides will be discussed in detail later. The studies using TAT-Cx43L2 are reported to be specific to hemichannel functions, indicating that there are differential regulatory pathways for GJ gating [114]. 

The Gap19 peptide, consisting of 9 amino acids within the Cx43L2 region, was described to alter Cx43, but not Cx40 hemichannels, preventing experimentally induced ischemia-reperfusion injury [115], and Cx43 hemichannel function in astrocytes [117]. The first Gap19 iteration, with a molecular weight of around 1100 Da and net charge of +3, was not attached with a specific internalization sequence, rather it was assumed the “KKFK” sequence permitted plasma membrane translocation as previously described [157]. Later, a TAT-bound Gap19 (TAT-19) was shown to have anti-inflammatory and neuroprotective effects [158]. TAT-Gap19 (Cx43), as well as TAT-GAP24 (same L2 region in Cx32), both block hemichannels reducing acetaminophen-induced liver injury [111], limiting Ca^2+^ activated hemichannel activation, and reducing arrhythmias in human heart tissues [159]. Recently, Coutinho et al. developed Xentry-Gap19, which is composed of Gap19 bound by the cell-penetrating “LCLRPV” peptide sequence, using Syndecan-4 for entry, potentially making it specific to cells expressing this receptor [118]. Xentry-Gap19 provides a greater hemichannel block than the Gap19 peptide alone, while not altering GJ signaling [118].

The Gap 19 and Cx43L2 peptides can directly bind the Cx43-CT. Ponsaerts et al. identified binding between the Cx43L2 peptide and the Cx43CT peptide containing the last 10 amino acids of Cx43, using surface plasmon resonance (SPR) analysis [114]. Further, Ponsaerts et al. determined that amino acids isoleucine (I130) and histidine (H126) are essential for peptide functions and hemichannel blockade. Interaction studies by Wang et al., using SPR, determined that GAP19, like Cx43L2, interacts with amino acids between 255–382 on the Cx43-CT [115]. SPR data suggest that GAP19 has a stronger interaction with the Cx43-CT^255∓382^ than the Cx43L2 peptide [115]. However, the Cx43CT protein used in these studies was longer (127 a.a.) than that used for the Ponsaerts SPR data (10 a.a.) [114], which may indicate different binding modalities or regions for Cx43-CT. A 9 a.a. peptide, Gap20, overlaps Gap19 and includes a.a. I130, but not H126, is biologically inactive and does not alter cellular signaling. Gap20 and another peptide, Gap22 (Cx40-L2 region), were ineffective against the phenylephrine-induced Ca^2+^ oscillations on rabbit arterial sections and vasoconstriction in rat mesenteric arteries [92,119]. Early et al. described a slight depolarization of the rat mesenteric arteries by Gap20, potentially as a result of high peptide concentrations of 300 μM [119]. While these experiments did not include the use of internalization sequences, their small size and charge could potentially allow for uptake through connexin hemichannels (as previously suggested [157]) which may account for these effects.

### 3.3. Connexin-Mimetic Peptides That Target the Carboxyl-Terminus

The intracellular CT regions of connexin proteins regulate a number of functions including, but not limited to, protein trafficking, assembly, and channel gating [160,161,162]. The connexin-CT is also known as a regulator of cell growth and differentiation that interacts with a number of proteins within the cell [162]. Given the critical connexin functions regulated by the CT, the region has been an attractive target for peptide therapies. 

#### 3.3.1. α. CT Peptides

The first biologically active peptide targeting the Cx43 CT was reported in 2005 by the Gourdie laboratory [121]. The alpha carboxyl terminus 1 (αCT1) peptide is a mimetic sequence to the last nine amino acids of the CT of Cx43 (RPRPDDLEI), attached to a 16-amino acid antennapedia sequence for cell internalization, linked to the peptide’s N-terminus [121]. Direct protein-peptide interactions have been demonstrated for Cx43 and αCT1, interacting with the Cx43-CT, Cx43-L2 domains, the second alpha-helical region of the Cx43-CT (H2; Figure 1), and the PDZ2 binding domain of zona occludins 1 (ZO1) [123]. 

As described above, treatment with αCT1 increases phosphorylation at Cx43-S368 in cultured HeLa cells [120,122]. Further, in cell culture, αCT1 has been shown to inhibit binding with its protein partner ZO1 [163]. Under physiological conditions, the PDZ2 domain of ZO1 has been shown to interact with the Cx43 CT and studies have demonstrated that ZO1 regulates the connexin to GJ transition, by slowing the rate at which hemichannels move into established GJ plaques [163,164,165]. Further, αCT1 treatment has been shown to increase GJ plaque size and GJ coupling, and decrease hemichannel activity [163]. At the organ level, αCT1 increases wound closure rates, reduces scarring, and reduces inflammation in the skin and retinal wounds [166]. In hearts, αCT1 has been proven effective in mitigating ischemia-reperfusion injury in murine models and has antiarrhythmic effects [123]. To date, the αCT1 peptide has shown promising results as a therapy for a variety of conditions at the pre-clinical and clinical levels, including cardiac disease, retinal injury, and wound healing; recently reporting successful phase II clinical trial data on the use of αCT1 for the treatment of venous leg ulcers and diabetic foot ulcers [48,53,167,168,169,170,171].

Several peptide modifications of αCT1 also exist. The αCT2 (also referred to as αCT11) [123] peptide consisting of the last nine amino acids of Cx43, but without an antennapedia sequence, and the αCT3 peptide consisting of the last 23 Cx43-CT amino acids with an antennapedia sequence were found to accelerate wound healing in mice [122]. Though, both peptides were less effective than αCT1. The αCT11 peptide has recently been shown to be cardioprotective against ischemia-reperfusion injury, preserving left ventricular developed pressure after injury associated with increases in Cx43-S368 phosphorylation [123].

#### 3.3.2. CT9 Peptide/CT10/TAT-Cx43 Peptides

Over the last decade, Leybaert and colleagues have published extensively on a peptide referred to as CT9, which is comprised of the same last 9 amino acids of the Cx43 CT found in the αCT11 peptide (Table 1) [101]. Supporting the earlier findings from the Gourdie laboratory’s work with αCT11, CT9 was found to block Cx43-ZO1 interactions and to trigger PKC-mediated Cx43-S368 phosphorylation [123,125]. In contrast to earlier works on αCT11, where several studies demonstrated the αCT peptides to block connexin hemichannels [122,172,173], CT9 is reported to acutely activate Cx32 and Cx43 hemichannels [101]. One important difference that may explain these discrepancies is that the αCT11 effect on hemichannels has been reported over hours or even days, whereas the effect of CT9 was measured over seconds to minutes. This could suggest a potential difference in short- and long-term peptide treatments to the hemichannel activation/inactivation axis. 

Several other studies have described the effects of two identical peptides covering the last 10 a.a. of the Cx43-CT, referred to as TAT-Cx43CT [114] and TAT-CT10 [115]. TAT-Cx43CT maintains hemichannel opening, allowing for the preservation of coordinated Ca^2+^ signaling in endothelial cells [114]. In their studies, Ponsaerts et al. demonstrated binding between Cx43CT and the PDZ2 domain of ZO-1 by SPR, with binding lost when using the TAT-Cx43CTΔI peptide that is lacking the last isoleucine [114]. However, ZO1-Cx43CT interaction did not alter peptide-linked hemichannel functions in these studies [114]. Both TAT-Cx43CT and TAT-CT10 peptides maintain hemichannel opening, in the presence of Cx43 hemichannel blocker peptides TAT-L2 (Cx43) and GAP19 [114,115]. This is thought to occur by peptide disruption of GAP19 binding the Cx43-CT [115]. Ponsaert et al. used SPR to demonstrate that Cx43-L2 and Cx43-CT peptides interact, suggesting that inhibition may occur by competition for binding sites on the Cx43-L2 region [114]. Studies by Wang et al., produced similar results with SPR analysis demonstrating binding between GAP19 peptide and the Cx43-CT (a.a. 255–382) and the Cx43-IL (a.a. 119–144). Although these studies did not directly demonstrate inhibition/ competition for binding by the TAT-CT10 peptide [115].

#### 3.3.3. TAT-Cx43 266–283 Peptide

SRC tyrosine kinases are involved in numerous cell functions, including cell metabolism, and are found in both proliferating and postmitotic cells. The protein exists in both a cellular form (cSRC) and as an oncogenic viral homolog (vSRC) [174]. Interaction between SRC and Cx43 has been demonstrated to occur at the Src homology 2 (SH2) and Src homology 3 (SH3) domains [175]. cSRC binding with the Cx43-CT results in the inhibition of cSRC. Active cSRC plays a role in perpetuating cancer cell proliferation including in glioma stem cells. Work from the Tabernero laboratory identified a Cx43 peptide spanning amino acids 266 to 283 (TAT-Cx43 266–283) that mimics the SRC SH3 binding domain along with a number of upstream residues [176]. This peptide contains a cell-penetrating TAT sequence allowing it to enter the intracellular space and bind cSRC, resulting in cSRC inactivation [176]. In cultured glioblastoma stem cells, obtained from patients, the application of TAT-Cx43 266–283 reduced cSRC activity and limited the glioblastoma phenotype [176]. TAT-Cx43 266–283 treatment in both in vivo mouse models and cultured human glioblastoma stem cells resulted in a reduction in glucose uptake and consumption. This effect was mediated by the inactivation of cSRC and a subsequent reduction in hexokinase-2 (HK-2), glucose-6-phosphate dehydrogenase (G6PD), and glucose transporter-3 (GLUT-3) proteins, all of which are involved in the proliferation of glioblastoma stem cells through the uptake and usage of glucose [128].

In neural progenitor cells, cSRC is involved in survival and proliferation via the epidermal growth factor (EGF) and fibroblast growth factor 2 (FGF-2) signaling pathways [177,178]. These progenitor cells, when treated with the Cx43 266–283 peptide in culture, exhibited impaired survival, proliferation, and differentiation rates due to cSRC inhibition. Interestingly, the TAT-Cx43 266–283 peptide increased rates of astrocyte proliferation in culture. The authors hypothesized that this is due to reductions in cSRC leading to a decrease in β-catenin expression by an unknown mechanism [129].

The interaction between Cx43 and cSRC has also been studied in the context of neuroinflammation. In response to neuroinflammation stimuli, TAT-Cx43 266–283 reduced cSRC levels and exhibited a neuroprotective effect [179]. In these studies, it was proposed that TAT-Cx43 266–283 induced cSRC inhibition interferes with the ability of activated astrocytes to mount an inflammatory response to neuronal injury. Astrocytic hemichannels, including Cx43, were inhibited by TAT-Cx43 266–283 indicating that cSRC mediates hemichannel activation [179]. 

#### 3.3.4. JM2 Peptide 

The Cx43 juxtamembrane 2 (JM2) connexin-mimetic peptide, published by Calder et al. in 2015, was designed to target amino acids 231–245 of the Cx43 CT, a known microtubule-binding domain [127]. Initial studies found that JM2 treatment significantly enhanced the Cx43-β-tubulin interaction thought to be important for Cx43 trafficking. Yet, paradoxically reduces cell surface Cx43 expression and GJ size [180]. Likewise, JM2 decreased GJ and hemichannel activity, as assessed by ATP release assay [127]. In a subsequent study, Rhett et al. speculated that decreased Cx43 channel activity resulted from enhanced Cx43-β-tubulin interactions, which led to Cx43 inappropriately ‘adhering’ to the microtubules as opposed to using them as a trafficking scaffold [23]. 

In vivo studies in mice and rats demonstrate that JM2 treatment reduces inflammatory cell infiltration following submuscular device implant [127], reduces tumor recurrence following solid tumor resection, and improves wound healing [180]. Further studies have shown that JM2 promotes tumor cell apoptosis in cultured B16F10 and Skov-3 tumor cells and improves the migration of cultured endothelial cells [180]. Recent research yielded promising results testing JM2 as a therapy for glioblastoma and there are currently groups looking to use JM2 loaded biodegradable nanoparticles to treat temozolomide resistant glioblastoma, with studies in preparation for clinical trials [181,182].

## 4. The Phosphorylation Phenomenon: Cx43-S368-Phosphorylation as a Key Factor in the Effect of Connexin Mimetic Peptides

One observation that bears mentioning in this review is the consistency with which Cx43 peptides induce PKC-associated Cx43-S368 phosphorylation (Table 1). Many of the Cx43 targeting peptides in this review, regardless of site-specificity to the EL, IL, or CT, appear to cause intrinsic changes in Cx43 phosphorylation. Phosphorylation of Cx43-CT is a key event in channel open/close probability but is also a significant modulator of protein-protein interactions that control protein trafficking and localization as well as non-GJ functions.

There is conflicting information on whether Cx43 PKC-phosphorylation leads to enhanced or reduced GJ coupling [81,158]. Phosphorylation of Cx43 by PKC is reported primarily at Cx43-S368, though five other sites are also reported to be altered, thus it is possible combinations of these phosphorylation sites may occur [81]. Phosphorylation of Cx43-S368 alters the random coil nature of the Cx43-CT and induces a more alpha-helical state, associated with reduced GJ formation and signaling [146,183,184]. Phosphorylation by PKC is also reported to alter the nature of Cx43 interactions with ubiquitin ligases and thus its degradation within the cell [185]. While PKC phosphorylation is generally associated with the closure of the gap junctions [146,186], several studies have found that peptide treatments (e.g., αCT1) enhance PKCε phosphorylation of Cx43, leading to increased gap junction stability and communication, but reduced hemichannel opening [125,163]. Similarly, the non-targeting peptides such as AAP10 and danegaptide, all increase Cx43-S368 phosphorylation, presumably through GPCR activation, which is reported to increase gap junction communication [65,72,78,82,83,84]. As noted by Jiang et al. the αCT1 and αCT11 peptides can interact with multiple regions on the Cx43 protein, and with other proteins, which may result in an alteration in accessibility of specific Cx43 amino acids. This may indicate that peptides altering the CT/L2 regions leaving the Cx43-S368 region more susceptible to phosphorylation [123]. Thus, it is possible that connexin mimetic peptide binding induces phosphorylation by altering the structure restraints of the Cx43-CT or by promoting phosphorylation combinations of the multiple suggested Cx43-PKC sites, which can result in both increased or decreased gap junction coupling [81].

The overall significance of the Cx43-PKC (S368) phosphorylation following peptide treatments remains to be fully elucidated. As described, many studies have found that peptide-induced Cx43-PKC phosphorylation was associated with improved outcomes, such as mitigated ischemic injury, reduced cellular proliferation in cancer, and improved wound healing. Thus, while highly speculative, it is possible that activating the PKC pathways may be considered cytoprotective.

## 5. Connexin Peptide Cross-Reactivity with Pannexins

The “Gap” peptides have shown promise of increased specificity to connexins by comparison to conventional channel blockers such as glycyrrhetinic acid [187]. However, there are serious concerns that they may target related channels and produce off-target effects. Recently, ^43^Gap27 and ^32^Gap24 have been found to block pannexin 1 (Panx1) channels at concentrations ranging from 200 μM to 2 mM [10]. This has led some to debate whether the connexin-mimetic peptides are truly sufficiently selective to allow for specific channel identification [10,187]

Pannexins form purine release channels that are structurally homologous to the connexins, containing four transmembrane domains, two EL, and cytoplasmic NT, IL, and CT but share little sequence homology. While connexins channels are known to form as hexamers, recent data has shown that Panx1 channels oligomerize as heptamers [188,189]. The pannexin channels function similarly to that described for connexin hemichannels and are known to release ATP as well as other molecules [190]. Several GJ blockers and non-specific channel blockers inhibit both connexin and pannexin channel release of ATP. This poses a complication with data interpretation, as measures of ATP release, used to assess connexin hemichannel function, are a shared output associated with alterations in pannexin channel activity. 

Connexin-mimetic peptides have demonstrated effects on pannexin channels. These peptides are known to attenuate ATP release and Ca^2+^ waves, associated with both connexin and pannexin channels. Both ^37,43^Gap27 and ^32^Gap24 can reversibly inhibit pannexin membrane currents [10]. ^37,43^Gap27 contains a sequence from an extracellular loop of Cx43, while ^32^Gap24 contains a sequence from the Cx32-IL. Interestingly, scrambled ^32^Gap24 showed similar levels of inhibition to its non-scrambled counterpart. Polyethyleneglycol 1500 (PEG 1500), an inert, biocompatible polymer inhibited pannexin channels similarly to the connexin-mimetic peptides. This along with the inhibitory activity of scrambled ^32^Gap24 peptides suggests inhibition of pannexins by mimetic peptides may function via steric block of the channel rather than peptide-protein interactions [10].

Multiple Panx1 mimetic peptides have been developed targeting sequences on both the intracellular and extracellular loops. Peptides E1a, E1b, E1c, E2a, and E2b target Panx1-EL sequences. Both E1b and E1c can inhibit Panx1 channels by more than 10%, with E1b having the strongest inhibitory effect. The peptide 10Panx1 contains the same sequence as E1b but is three amino acids shorter. Both 10Panx1 and E1b inhibit Panx1 channels to a similar extent [10]. Other Panx1 peptide inhibitors include PxIL2P that targets the Panx1-IL2 region developed by Isakson et al. [191,192], and an interfering peptide Panx Y308, which has a target sequence between a.a. 305 and 318 on the Panx 1-CT developed by the Thompson lab [193,194]. 

The peptide 10Panx1 inhibits Panx1 currents compared with its scrambled sequence, suggesting specificity [195,196,197]. However, 10Panx1 has also been found to inhibit Cx46 channels [10]. PxIL2P reportedly targets a Panx1 sequence necessary for Panx1 activation by alpha 1 andenosine receptor signaling [192]. Application of PxIL2P significantly decreases phenylephrine-induced smooth muscle cell constriction in C57BL/6 mice. These effects were similar to those observed in smooth muscle cell-specific Panx1 knockout mice, suggesting that PxIL2P is at least somewhat specific to Panx1 channels [192]. Panx Y308 was created specifically to target the Panx1 Src family kinases (SFK) consensus-like sequence between Panx1 305 and 318. This sequence contains a site at tyrosine Y308 where SFK is believed to phosphorylate Panx1 and causes channel opening in anoxic conditions. Panx Y308 has been shown to attenuate anoxic depolarization of Panx1 [193]. 

## 6. Concluding Remarks—Clinical Applications and Future Approaches

In the last 25 years, several encouraging lines of evidence have demonstrated that connexin targeting peptides can improve disease outcomes in pre-clinical models of disease. Connexin proteins are attractive therapeutic targets given their prevalence in disease, although approaches to target connexins are complicated due to the ubiquitous distribution of the proteins in most tissues. Inappropriately reducing the expression of connexins or inhibiting GJ functions can be lethal. Alterations or mutations in vasculature-associated connexins, such as Cx37 and Cx40, lead to disturbances in blood vessel relaxation and have knock-on effects in other systems leading to alteration in renin secretion and increases in blood pressure. Genetic connexin mutations in humans are rare, but several have been identified. Mutations in Cx43 have been linked with cardiovascular disease [198,199,200,201,202], and Cx26 mutations lead to sensorineural hearing loss [203,204] and keratoderma [205,206]. As a result, systemically targeting reductions in connexin expression provides fairly limited options due to the potential for side effects. However, a greater understanding of disease-associated, and potentially redundant mechanisms may help provide avenues for future therapeutic intervention. 

At present, the instability of peptides severely limits their therapeutic potential. Most peptides have a half-life of seconds to minutes and are quickly degraded by changes in pH, shifts in temperature, and peptidases, making systemic delivery challenging at best [207]. Chen et al. added the lipid-alkyl groups (C12-Laa) moieties to extended GAP27 peptides increasing half-life from 145 min to around 350 min, which could increase their therapeutic viability [109]. Protecting the peptides from peptidases is another potential option, and can be accomplished through peptide cyclization. This has the advantage of decreasing enzyme breakdown but may also limit targeting of the peptides by limiting access to active sites and blocking structural-based interactions [123]. Cyclization may also be limited in peptides containing cysteines, which are found in connexin CT and EL, as they must be removed for the production of disulfides bonds during peptide cyclization. 

Likely, future therapeutic approaches may also require systemic delivery by encapsulation of peptides (such as in exosomes [208]), in a process that allows for tissue-specific, and intracellular targeting of the connexin proteins. There is still a general lack of detail on the overall peptide mechanisms that produce their effects. Greater examination of the molecular pathways underlying the peptide effects could help define more specific therapeutics directed at the connexin proteins and their functions and may also allow for the development of peptide-based therapeutics such as small molecule discovery or single domain antibodies targeting these regions.
